# Cardiovascular adverse events associated with BRAF versus BRAF/MEK inhibitor: Cross‐sectional and longitudinal analysis using two large national registries

**DOI:** 10.1002/cam4.3938

**Published:** 2021-05-13

**Authors:** Avirup Guha, Prantesh Jain, Michael G. Fradley, Daniel Lenihan, Jahir M. Gutierrez, Chhavi Jain, Marcos de Lima, Jill S. Barnholtz‐Sloan, Guilherme H. Oliveira, Afshin Dowlati, Sadeer Al‐Kindi

**Affiliations:** ^1^ Harrington Heart and Vascular Institute University Hospitals Case Western Reserve University Cleveland OH USA; ^2^ Division of Hematology and Medical Oncology University Hospitals Cleveland Medical Center Seidman Cancer Center at Case Comprehensive Cancer Center Case Western Reserve University Cleveland OH USA; ^3^ Cardio‐Oncology Program Division of Cardiology University of Pennsylvania Philadelphia PA USA; ^4^ Cardio‐Oncology Center of Excellence Division of Cardiology Washington University in St Louis St. Louis MO USA; ^5^ Layer6 Inc. Toronto Canada; ^6^ Lerner Research Institute Cleveland Clinic Case Comprehensive Cancer Center Cleveland OH USA; ^7^ Department of Population and Quantitative Health Sciences Case Comprehensive Cancer Center Case Western Reserve University School of Medicine Cleveland OH USA; ^8^ Division of Cardiovascular Sciences Cardio‐Oncology Program University of South Florida Tampa General Hospital and Moffitt Cancer Center Tampa FL USA

**Keywords:** BRAF inhibitors, BRAF/MEK inhibitors, cardiotoxicity, cardiovascular adverse events, FAERS, Marketscan, pharmacoepidemiology

## Abstract

**Background:**

Cardiovascular adverse events (CVAEs) associated with BRAF inhibitors alone versus combination BRAF/MEK inhibitors are not fully understood.

**Methods:**

This study included all adult patients who received BRAF inhibitors (vemurafenib, dabrafenib, encorafenib) or combinations BRAF/MEK inhibitors (vemurafenib/cobimetinib; dabrafenib/trametinib; encorafenib/binimetinib). We utilized the cross‐sectional FDA’s Adverse Events Reporting System (FAERS) and longitudinal Truven Health Analytics/IBM MarketScan database from 2011 to 2018. Various CVAEs, including arterial hypertension, heart failure (HF), and venous thromboembolism (VTE), were studied using adjusted regression techniques.

**Results:**

In FAERS, 7752 AEs were reported (40% BRAF and 60% BRAF/MEK). Median age was 60 (IQR 49–69) years with 45% females and 97% with melanoma. Among these, 567 (7.4%) were cardiovascular adverse events (mortality rate 19%). Compared with monotherapy, combination therapy was associated with increased risk for HF (reporting odds ratio [ROR] = 1.62 (CI = 1.14–2.30); *p* = 0.007), arterial hypertension (ROR = 1.75 (CI = 1.12–2.89); *p* = 0.02) and VTE (ROR = 1.80 (CI = 1.12–2.89); *p* = 0.02). Marketscan had 657 patients with median age of 53 years (IQR 46–60), 39.3% female, and 88.7% with melanoma. There were 26.2% CVAEs (CI: 14.8%–36%) within 6 months of medication start in those receiving combination therapy versus 16.7% CVAEs (CI: 13.1%–20.2%) among those receiving monotherapy. Combination therapy was associated with CVAEs compared to monotherapy (adjusted HR: 1.56 (CI: 1.01–2.42); *p* = 0.045).

**Conclusions and Relevance:**

In two independent real‐world cohorts, combination BRAF/MEK inhibitors were associated with increased CVAEs compared to monotherapy, especially HF, and hypertension.

## Graphical abstract

In two independent real‐world cohorts, combination BRAF/MEK inhibitors were associated with increased cardiovascular adverse events compared to monotherapy, especially HF, and hypertension.

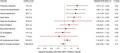

## 1 INTRODUCTION

Targeted anti‐cancer agents have significantly impacted the cancer treatment landscape. Recent advances in melanoma, colon, and lung cancer have prompted the development of a new class of targeted therapeutics: BRAF inhibitors.^1^ Based on positive clinical studies, vemurafenib was the first BRAF inhibitor to be approved by the Food and Drug Administration (FDA) as single‐agent therapy for metastatic melanoma. Further research has shown that patients with metastatic disease may become resistant to single‐agent therapy, and BRAF agents may not be as effective alone.^2^ Thus, a new class of drugs that target MEK downstream from the BRAF in the signaling cascade was recommended as a novel strategy to overcome resistance and add to efficacy when used combined with BRAF inhibitors. Multiple combinations of BRAF/MEK inhibitors have recently found to be highly efficacious for the use in metastatic melanoma, metastatic non‐small cell lung cancer, and metastatic colon cancer,^3^, ^4^, ^5^, ^6^, ^7^, ^8^, ^9^ which include vemurafenib (BRAF)/cobimetinib (MEK), dabrafenib (BRAF)/trametinib (MEK), and encorafenib (BRAF)/binimetinib (MEK).

Several studies have reported drug‐related cardiovascular adverse events (CVAE) with BRAF monotherapy; however, the incidence of CVAEs may be higher when used in combination with MEK inhibitors.^10^ The most common CVAEs are asymptomatic reductions in left ventricular ejection fraction (LVEF), symptomatic heart failure (HF), hypertension, QT interval prolongation, and atrial arrhythmias.^11^ The incidence of LVEF reduction and hypertension was noted to be around 5%–11% and 10%–15%, respectively.^7^, ^9^ In a recent meta‐analysis, it was noted that the BRAF/MEK combination was associated with an increased risk of pulmonary embolism (relative risk [RR] = 4.36, confidence interval [CI] = 1.23–15.45), decreased LVEF (RR 3.72 [CI = 1.74–7.95]) and hypertension (RR 1.49 [CI = 1.12–1.98]).^11^

Thus far, the reported data are from clinical trials, which included a small number of events, and real‐world and pharmacovigilance data are not yet available. In this manuscript, we used two real‐world data sources–FDA’s Adverse Event Reporting System (FAERS) and Truven Health Analytics/IBM MarketScan Commercial Claims database to study the reporting of CVAEs for patients on BRAF inhibitors alone versus combination BRAF/MEK inhibitors, along with associated risk factors and outcomes.

## 2 METHODS

### 2.1 Data source

A cross‐sectional analysis was done using the FAERS dataset due to its design. The FAERS is a pharmacovigilance system maintained by the FDA that includes AE reports that are voluntarily submitted. This registry provides data for post‐marketing safety surveillance and adheres to the international safety reporting guidance issued by the International Conference on Harmonization (ICH E2B), where all adverse events are coded using terms in the Medical Dictionary for Regulatory Activities.^12^ We searched the FAERS registry from January 1, 2011, to September 30, 2019, for adverse events reported for BRAF inhibitors (vemurafenib, dabrafenib, encorafenib) or a combination of BRAF/MEK inhibitors (vemurafenib (BRAF)/cobimetinib (MEK), dabrafenib (BRAF)/trametinib (MEK), and encorafenib (BRAF)/binimetinib (MEK)) among patients ≥18 years of age, excluding reports that did not specify patient age, gender, and type of cancer.

Longitudinal analysis was done using Truven Health Analytics/IBM MarketScan Commercial Claims, and Encounters Database was available from January 1, 2011, to December 30, 2018. The Truven Health Analytics/IBM MarketScan Database consists of de‐identified outpatient, inpatient, and pharmaceutical claims using over 50 million privately insured patients each year obtained from 150 large employer‐sponsored health insurance plans in all 50 states. We queried the entire dataset for the prescription of drugs listed above using the International Classifications of Disease version 9 and 10 (*ICD*‐*9 CM* and *ICD*‐*10 CM)* codes and created a subset that included patients with at least one prescription of BRAF monotherapy or combination BRAF/MEK. The enrolment of at least 90 days before drug initiation was required to ensure adequate risk factor analysis for adjudicated events.

This study is compliant with the Declaration of Helsinki, and given the retrospective nature of the data‐informed consent was not required. The data that support the findings from the FAERS part of this study are openly available in raw format at https://bit.ly/37cHoXC. The data that support the findings of the Truven Health Analytics/IBM MarketScan part of the study is available from IBM. Restrictions apply to the availability of these data, which were used under license for this study. Thus, this data cannot be shared directly by the authors.

### 2.2 Outcomes

The primary outcome of this study is the proportion of CVAEs by dataset, classified into hypertension, atrial fibrillation, HF and reduced LVEF, QT prolongation/ventricular arrhythmia, pulmonary embolism/deep vein thrombosis/venous thromboembolism, myocardial infarction (MI), and stroke. All‐cause mortality stratified by CVAE event is a secondary outcome in the Truven Health Analytics/IBM MarketScan dataset. Mortality, hospitalization, and major adverse cardiovascular events (MACE) among adverse events were secondary outcomes in the FAERS registry. MACE was defined as any CVAEs or mortality.

In the FAERS dataset, reported adverse events are a combination of symptoms and diagnoses that are classified into 25 subcategories according to ICH E2B. Specific cardiovascular disorders were identified through keywords detailed in Table S1.

In the Truven Health Analytics/IBM MarketScan dataset, the outcomes were identified in the inpatient, outpatient, and provider claims files using *ICD*‐*9 CM* and *ICD*‐*10 CM* codes. The National Cancer Institute (NCI) Macro,^13^ designed to identify comorbidities, was repurposed to identify primary outcomes and other diagnoses.^14^ The first date of diagnosis of the outcomes was tabulated and adjudicated as an event if the patient did not have the outcome before the first prescription of the drug. QT prolongation/ventricular arrhythmia could not be identified with certainty in the Truven Health Analytics/IBM MarketScan dataset and was only studied using FAERS. All the *ICD*‐*9 CM* and *ICD*‐*10 CM* of the outcomes are listed in Table S1.

### 2.3 Covariates

#### 2.3.1 FAERS

The FAERS event report form for each patient consists of a case identification number, suspected medication, reason for use, adverse reactions, nature of the event (i.e., serious vs. non‐serious), outcomes (hospitalization, death, and other outcomes), weight, event date, the pharmaceutical company, reporter (e.g., health care professional, consumer, a pharmaceutical company or unknown), concomitant medications, and country of event.^15^ Age was tiered into 18–39, 40–59, 60–79, and ≥80 years.

#### 2.3.2 Truven Health Analytics/IBM MarketScan

Claims within 5 years prior to the use of the drug therapy were used to generate the NCI comorbidity index. Death was available as an outcome before January 1, 2016. However, for performing competing risk analysis, death had to be defined using other variables, which are indicative of death (Supplemental methods).

### 2.4 Statistical analysis

#### 2.4.1 FAERS and Truven Health Analytics/IBM MarketScan

All continuous variables were assessed with the Shapiro‐Wilk test and visual histogram check for normality. Descriptive statistics using the chi‐squared test for categorical variables and Wilcoxon test/Student's *t*‐test for continuous variables were used to differentiate the demographic and clinical characteristics of those who received combination therapy versus monotherapy.

#### 2.4.2 FAERS

Multivariable polytomous logistic regression controlling for age, sex, cancer type, and data reporting source was performed to present the reported odds‐ratio of primary CVAE outcomes in combination therapy versus monotherapy.^16^

#### 2.4.3 Truven Health Analytics/IBM MarketScan

Univariate comparisons between BRAF monotherapy versus combination therapy were performed using the Kaplan–Meier method, and comparisons were made using the log‐rank (Mantel–Cox) test. A multivariable Cox regression model was used to evaluate the risk of CVAEs in combination versus monotherapy in our entire cohort. A multivariable adjustment was made for age (groups ≤35, 35 to 50, >50 to 64, and ≥65 years of age), year of cancer diagnosis (2011–2014, and 2015–2018), NCI comorbidity score (0 and >0), prior hypertension, insurance (Preferred Provider Organization [PPO], Health Maintenance Organization [HMO], and other), and geographical region (northeast, north‐central, south, west, and unknown). Given the high mortality rates observed with cancers treated with MEK or BRAF inhibitors, we also performed a sensitivity analysis using multivariable Fine and Gray regression to evaluate for each of the CVAEs, using death as a competing event, and comparisons were made using the Gray K‐sample test. Cause‐specific multivariable hazards ratio (HR) was presented.

The alpha for the analysis was set at 0.05. The classification of adverse events and hierarchical clustering analysis in FAERS was performed in Python 3.8.1, and the remaining statistics were performed using SAS 9.4.

## 3 RESULTS

**FIGURE 1:**
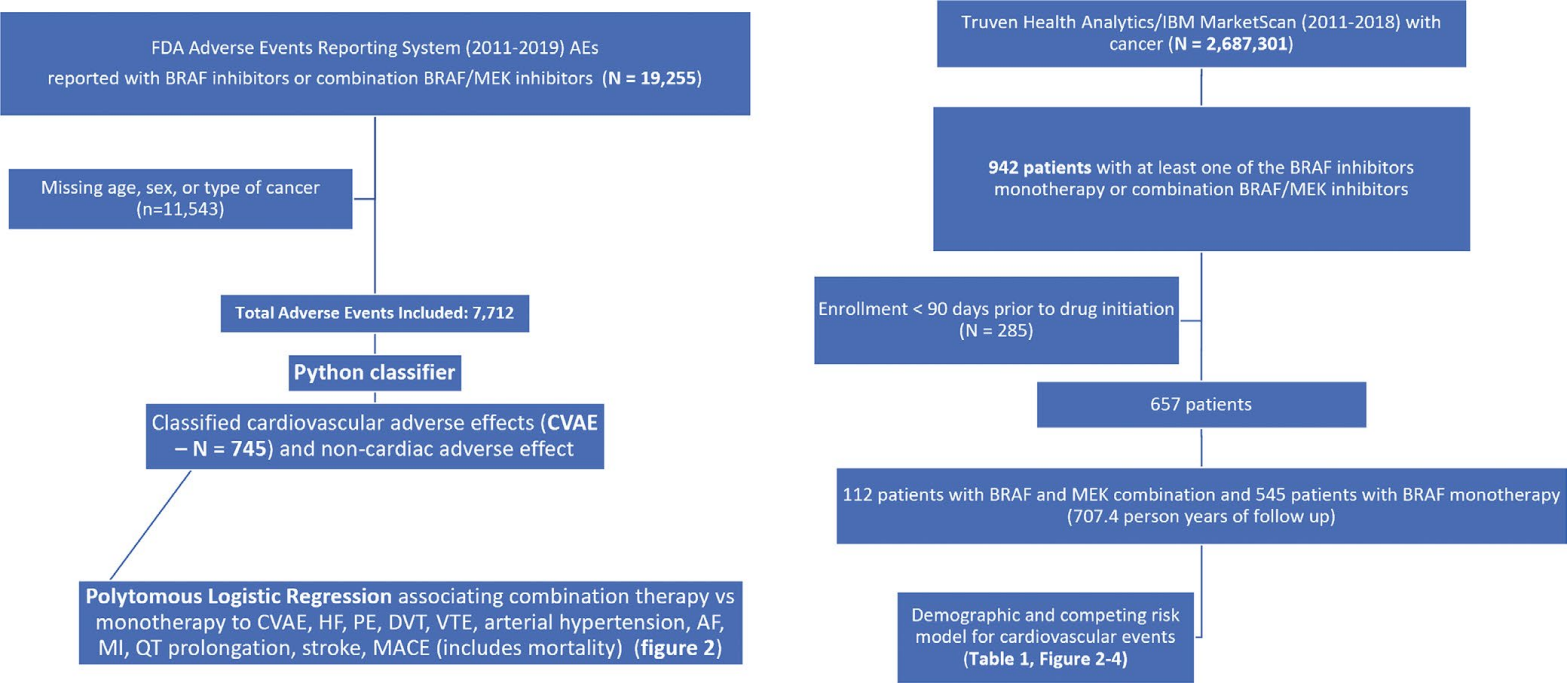
Consort diagram

The overall study design is presented as a consort diagram in Figure 1.

### 3.1 FAERS (cross‐sectional analysis)

#### 3.1.1 Demographics

Baseline characteristics of the FAERS population are presented in Table 1. Overall, 7712 adverse events were reported between 2011 and 2019, with the highest number of adverse events reported in 2016 (16.9% of all reports). Among reported adverse events, 4659 (60.4%) were noted in those receiving combination BRAF/MEK therapy compared to 3053 (39.6%) receiving monotherapy. The median age of those reporting adverse events was 60 years (IQR =49–69), and 44.9% were females. The most common source of the report were healthcare professionals (84.1%), followed by drug manufacturers (14.7%). Hospitalization was required for 46.2% of these adverse events, and 1427 (18.5%) died of adverse events.

**TABLE 1:** Characteristics of all adverse events with BRAF/MEK inhibitor combination therapy versus monotherapy with BRAF inhibitors in FDA Adverse Event Dataset

	Combination therapy (*N* = 4659)	Monotherapy (*N* = 3053)	*p*‐value
Female, *N* (%)	2118 (45.5)	1367 (44.8)	0.56
Age, *N* (%)			0.0002
18–39 years	500 (10.7)	291 (9.5)	
40–59 years	1738 (37.3)	1154 (37.8)	
60–79 years	2177 (46.7)	1378 (45.1)	
≥80 years	244 (5.3)	230 (7.5)	
Type of cancer^a^			—
Melanoma, *N* (%)	4406 (94.6)	2947 (96.5)	<0.0001
Non‐small cell lung cancer, *N* (%)	321 (6.9)	106 (3.5)	<0.0001
Colon cancer, *N* (%)	34 (0.7)	38 (1.3)	0.02
Year of reporting, *N* (%)			<0.0001
2011–2014	674 (14.5)	1862 (61.0)	
2015–2019	3985 (85.5)	1191 (39.0)	
Reporting source, *N* (%)			<0.0001
Consumer	0 (0)	15 (0.5)	
Pharmaceutical company	4004 (85.9)	2460 (80.6)	
Healthcare professional	642 (13.8)	511 (16.8)	
Other	13 (0.3)	67 (2.2)	
Reaction type, *N* (%)			<0.0001
Serious	4271 (91.7)	2640 (86.5)	
Non‐serious	388 (8.3)	413 (13.5)	
Hospitalized, *N* (%)	2378 (51.0)	1180 (38.7)	<0.0001
Died, *N* (%)	627 (20.0)	853 (18.3)	0.053
Specific cardiac events, *N* (%)^b^	380 (8.2)	187 (6.1)	0.0008
Pulmonary embolism^c^	56 (14.6)	19 (9.9)	0.03
Venous thromboembolism^c^	65 (17.2)	23 (12.5)	0.03
Arterial hypertension^c^	62 (16.4)	23 (12.5)	0.047
Heart failure^c^	110 (29.0)	44 (23.4)	0.03
Deep vein thrombosis^c^	14 (3.6)	6 (3.1)	0.19
Atrial fibrillation^c^	62 (16.2)	33 (17.7)	0.08
Myocardial infarction^c^	44 (11.5)	27 (14.6)	0.01
QT prolongation^c^	65 (17.2)	45 (24.0)	0.06
Stroke^c^	9 (2.3)	8 (4.2)	0.09

^a^ Few events have more than 1 reported cancer.

^b^ Hypertension, atrial fibrillation, HF and reduced LVEF, QT prolongation/ventricular arrhythmia, pulmonary embolism/deep vein thrombosis/venous thromboembolism, myocardial infarction, and stroke.

^c^ The proportions reflect that among CVAEs.

Among those who were on BRAF inhibitor monotherapy, the related adverse events were reported in vemurafenib (90.0%), dabrafenib (9.9%), and encorafenib (0.1%). Among those experiencing adverse events with combination therapy, dabrafenib (BRAF)/trametinib (MEK), vemurafenib (BRAF)/cobimetinib (MEK), and encorafenib (BRAF)/binimetinib (MEK) were the responsible drug combinations in 73.8%, 24.9%, and 1.3%, respectively.

In a multivariable logistic regression model adjusted for age, gender, cancer type, and reporting source, all adverse events resulting in hospitalization (ROR = 1.64 (CI = 1.49–1.81); *p* < 0.0001) were significantly associated with combination therapy in comparison to monotherapy. However, there was no difference in all adverse events resulting in mortality (reporting odds ratio [ROR] = 0.90 (CI = 0.80–1.01); *p* = 0.08) when comparing combination therapy to monotherapy.

#### 3.1.2 Cardiovascular adverse events

**FIGURE 2:**
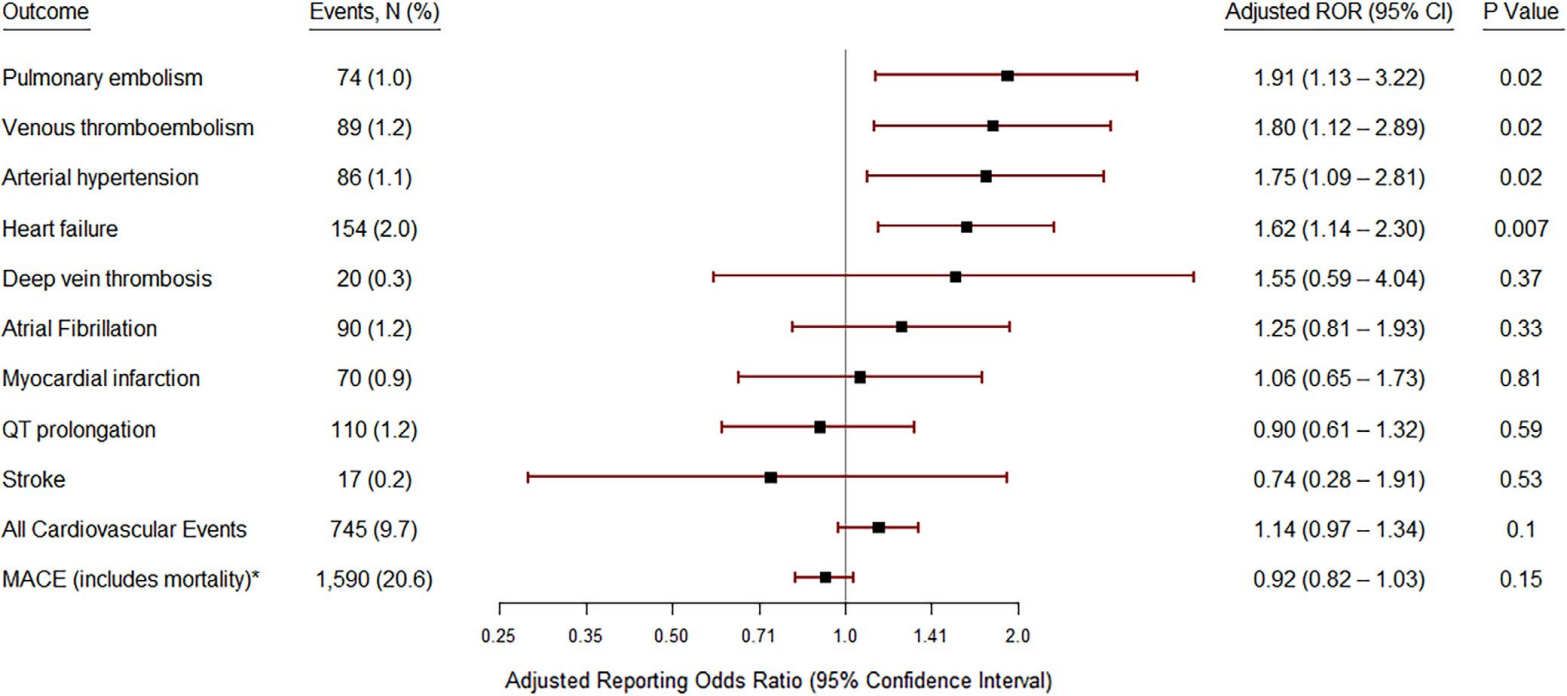
Cardiovascular Events and adjusted reporting odds ratio for MEK and BRAF combination therapy events versus monotherapy with BRAF inhibitors in the FDA Adverse Event Dataset. It was adjusted for age, gender, cancer type, and reporting source. MACE (major adverse cardiovascular event) is defined as any cardiac event in the list reported here or mortality

All cardiovascular adverse events represented 567 (7.4%) of all reported adverse events. The median age was 67 years (IQR = 57–74), and 45.8% were females. Hospitalization was required in 64.8% of cases, and 18.4% died. The most common adverse event was HF 154 (27.2%). In a multivariable logistic regression model adjusted for age, gender, cancer type, and reporting source, HF (reporting odds ratio [ROR] = 1.62 (CI =1.14–2.30); *p* = 0.007), arterial hypertension (ROR = 1.75 (CI = 1.12–2.89); *p* = 0.02) and venous thromboembolism (ROR =1.80 (CI = 1.12–2.89); *p* = 0.02) were significantly associated with combination therapy in comparison to monotherapy. Type of events and multivariable odds ratio comparing the likelihood of occurrence of various CVAEs with the combination versus monotherapy is presented in Figure 2.

In a multivariable logistic regression model adjusted for age, gender, cancer type, and reporting source, there was no difference in CVAEs resulting in hospitalization (ROR = 1.15 (CI = 0.79–1.67); *p* = 0.46) or CVAEs resulting in mortality (reporting odds ratio [ROR] = 0.89 (CI =0.56–1.39); *p* = 0.60) when comparing combination therapy to monotherapy.

### 3.2 Truven Health Analytics/IBM MarketScan (longitudinal analysis)

#### 3.2.1 Demographics

Baseline characteristics of those receiving BRAF monotherapy compared to BRAF/MEK combination therapy in the Truven Health Analytics/IBM MarketScan database are presented in Table 2. Overall, 657 patients with cancer representing 707.4 person‐years of follow‐up met the study criteria. The Median follow‐up time was 207 days (IQR 85–476). BRAF/MEK combination versus BRAF monotherapy was utilized in 112 (17.1%) versus 545 (82.9%) patients. The median age was 53 years (IQR 46–60), and 39.3% were females. The most common malignancy was melanoma (88.7%), and most patients had PPO insurance (63.6%). The overall use of anthracycline and radiotherapy in this cohort before the use of MEK or BRAF/MEK inhibitor was 0.5% and 7.9%, respectively.

**TABLE 2:** Characteristics of BRAF/MEK inhibitor combination therapy versus monotherapy with BRAF inhibitors in Truven Health Analytics/IBM MarketScan Dataset

	Combination therapy (*N* = 112)	Monotherapy (*N* = 545)	*p*‐value
Female, *N* (%)	48 (42.9)	210 (38.5)	0.39
Age, *N* (%)			0.69
<35 years	8 (7.1)	38 (7.0)	
35–50 years	42 (37.5)	182 (33.4)	
>50–65	62 (55.4)	325 (59.6)	
Type of cancer, *N* (%)			—
Melanoma	107 (95.5)	476 (87.3)	
Non‐small cell lung cancer	8 (1.5)	0	
Colon cancer	23 (4.2)	3 (2.7)	
Others	38 (7.0)	2 (1.8)	
Year of diagnosis, *N* (%)			<0.0001
2011–2014	11 (9.8)	476 (87.3)	
2015–2018	101 (90.2)	69 (12.7)	
Median follow up, days (median, IQR)	130 (51–363)	234 (95–506)	0.0003
NCI comorbidities, *N* (%)			<0.0001
0	72 (64.3)	444 (81.5)	
≥1	40 (35.7)	101 (18.5)	
Pre drug start comorbidities, *N* (%)			
Hypertension	28 (17.4)	95 (25.0)	0.06
Diabetes	13 (11.6)	40 (7.3)	0.13
Coronary artery disease	4 (3.6)	6 (1.1)	0.06
Congestive heart failure	3 (2.7)	8 (1.5)	0.18
Atrial fibrillation	2 (1.8)	11 (2.0)	0.29
Stroke	6 (5.4)	35 (6.4)	0.67
Venous thromboembolism	9 (8.0)	16 (2.9)	0.01
Anthracycline use, *N* (%)	0	3 (0.6)	—
Radiation use, *N* (%)	29 (4.2)	23 (25.9)	<0.0001

#### 3.2.2 Cardiovascular events

**FIGURE 3:**
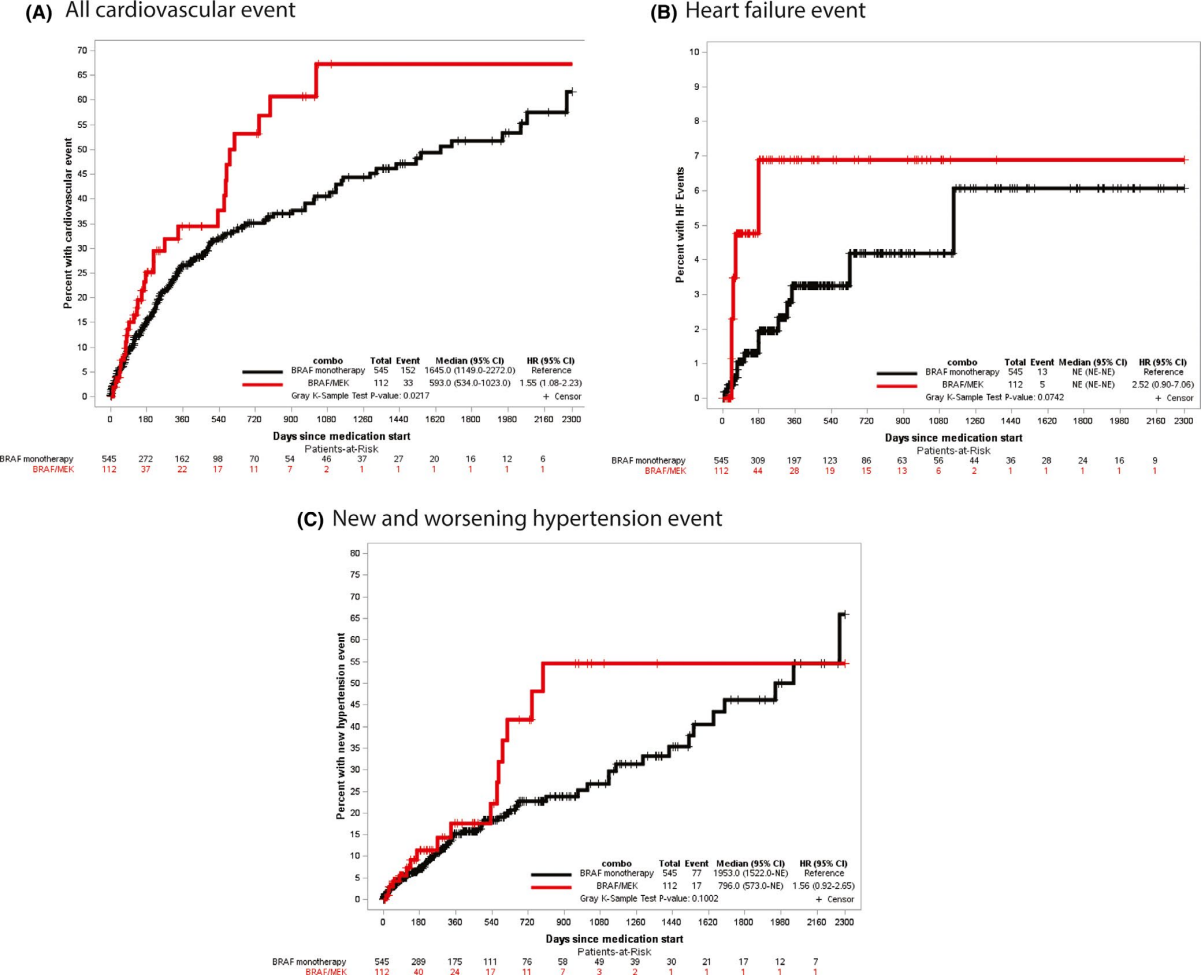
Competing risk analysis of (A) cardiovascular events, (B) heart failure, and (C) new or worsening hypertension in those receiving MEK and BRAF combination therapy versus monotherapy with BRAF inhibitors from Truven Health Analytics/IBM MarketScan dataset

Overall, 185 (28.2%) patients had incident CVAEs during the follow‐up period. There were 26.2% CVAEs (CI: 14.8%–36%) within 6 months of medication start in those receiving combination therapy versus 16.7% CVAEs (CI: 13.1%–20.2%) among those receiving monotherapy (Table 3). In a Cox regression model, the risk of all CVAEs was not statistically different among combination versus monotherapy (HR: 1.45 (CI: 0.99–2.13). However, when accounting for competing risk of death and adjusting for NCI comorbidity index, year of diagnosis, insurance status, and radiation therapy, combination therapy was associated with a higher risk of CVAEs compared to monotherapy (Adjusted HR: 1.56 (CI: 1.01–2.42); Gray K‐Sample *p*‐value: 0.045, Figure 3A). During follow‐up, 13 patients in the BRAF monotherapy arm had HF (6‐month rate of 1.9% (CI: 1.0–3.9%)) compared to 5 patients in the BRAF/MEK combination arm (6‐month rate of 6.9% (CI: 2.8–16.7%); Figure 3B). HTN event was noted in 73 in the BRAF monotherapy arm versus 19 patients in the BRAF/MEK combination arm (6‐month rate 7.1% (CI: 5.1%–10.0%) versus 11.3% (CI: 5.7–22.7%); Figure 3C).

**TABLE 3:** Hazards Ratio for events in BRAF/MEK inhibitor combination therapy versus monotherapy with BRAF inhibitors in Truven Health Analytics/IBM MarketScan dataset

Event	Events at 6 months in combination versus monotherapy (events (%))^b^	Cox Hazards Ratio	Competing risk Hazards Ratio^a^	Multivariable Cox Hazard Ratio^c^	*p*‐value^d^
Heart failure	5 (4.8%) versus 13 (1.4%)	2.52 (0.89–7.11)	2.66 (0.96–7.38)	—	0.07/0.052
Stroke	6 (9.2%) versus 28 (6.2%)	1.37 (0.75–2.51)	1.50 (0.85–2.67)	—	0.30/0.17
Pulmonary embolism	1 (1.8%) versus 9 (2.3%)	1.08 (0.32–3.68)	1.13 (0.33–3.88)	—	0.90/0.83
Deep vein thrombosis	1 (2.2%) versus 6 (2.3%)	1.11 (0.33–3.77)	1.25 (0.37–4.25)	—	0.87/0.72
Venous thromboembolism	1 (2.2%) versus 9 (2.3%)	0.94 (0.33–2.67)	1.13 (0.33–3.86)	—	0.90/0.83
New arterial hypertension	8 (11.1%) versus 32 (6.3%)	1.56 (0.92–2.65)	1.66 (0.99–2.79)	—	0.10/0.06
Atrial fibrillation	2 (2.9%) versus 8 (3.3%)	0.80 (0.18–3.49)	0.86 (0.20–3.77)	—	0.77/0.87
All cardiovascular events^e^	19 (26.2%) versus 73 (16.7%)	1.45 (0.99–2.13)	1.55 (1.08–2.23)	1.56 (1.01–2.42)^f^	0.06/0.045

^a^ Competing risk of mortality.

^b^ Calculated using the lifetable method of events/number at risk.

^c^ Only if the univariable model significant.

^d^ Log‐rank test/Gray K‐Sample test of univariable Cox model unless a multivariable model is used. In that case, a multivariable *p*‐value is presented.

^e^ Composite of heart failure, stroke, venous thromboembolism, hypertension, and atrial fibrillation.

^f^ Adjusted for the year of diagnosis, NCI comorbidity index, type of insurance since those were significantly different in the cohort.

Cox regression HR, cause‐specific HR accounting for competing risk, and 6‐month rate of an event of combination therapy versus monotherapy for individual events of HF, ischemic stroke, PE, DVT, VTE, new or worsening arterial hypertension, AF, and all CVAEs is presented in Table 2. The competing risk model plots for combination versus monotherapy are presented for stroke, VTE, and AF is shown in Figure S1A–C. None of the individual events achieved statistical significance, but HF (HR: 2.66, Gray K‐Sample *p*‐value: 0.052) and new or worsening arterial hypertension (HR: 1.66, Gray K‐Sample *p*‐value: 0.06) tended toward significance.

**FIGURE 4:**
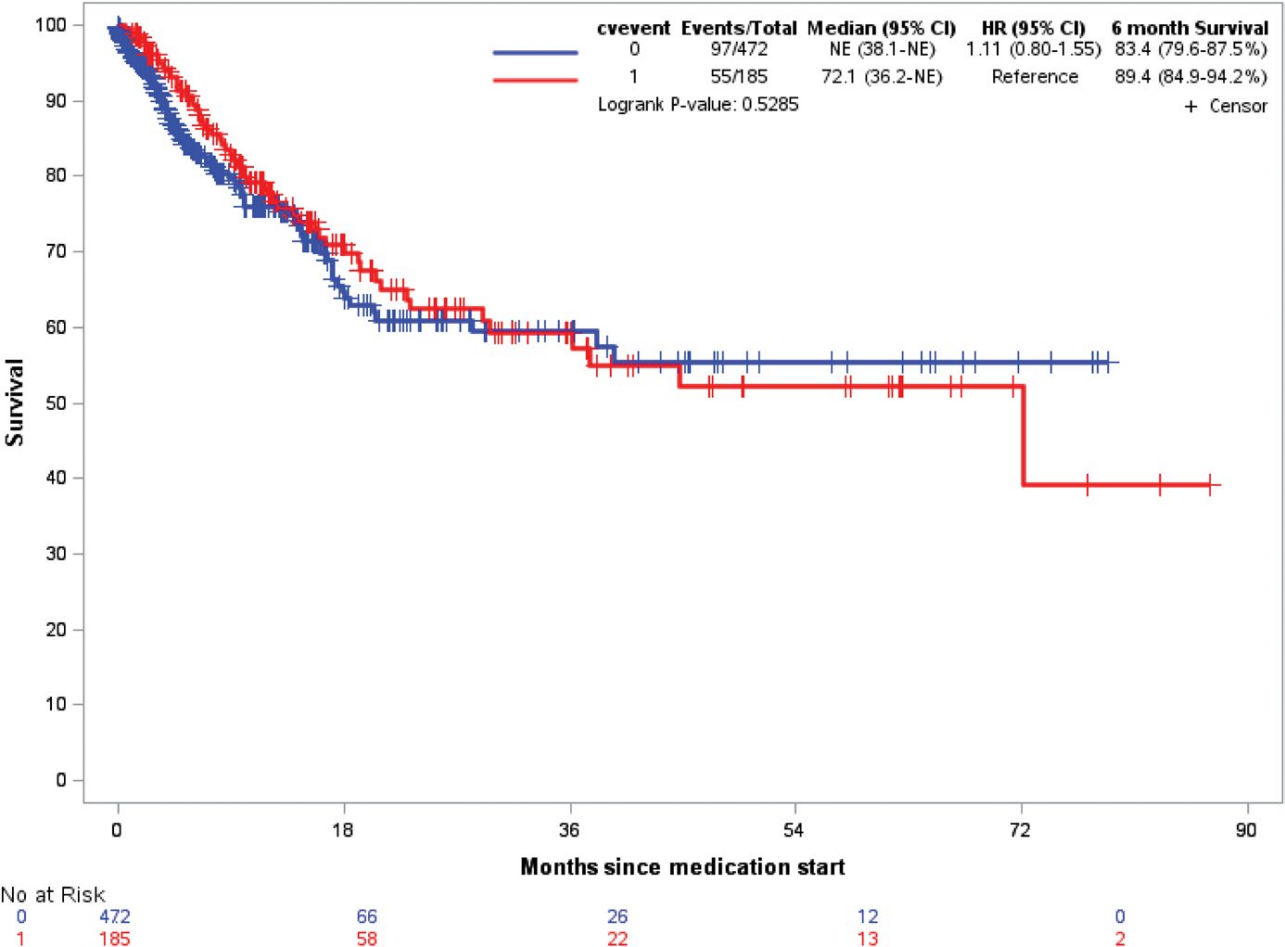
Kaplan–Meier Analysis of all‐cause mortality in those with CVAE versus those without CVAE among patients who undergo therapy with either MEK and BRAF combination therapy or monotherapy with BRAF inhibitors from Truven Health Analytics/IBM MarketScan dataset

There was no difference in all‐cause mortality among those who do not have any CVAEs versus those who have a CVAE, regardless of combination or monotherapy (HR: 1.11, Log‐Rank *p*‐value: 0.53; Figure 4).

## 4 DISCUSSION

In this study spanning 8 years using a large national pharmacovigilance database and a longitudinal cohort of privately insured patients, BRAF/MEK combination therapy was adversely associated with cardiovascular events compared to BRAF monotherapy. In a cross‐sectional pharmacovigilance analysis, HF, VTE, and arterial hypertension were significantly associated with combination therapy. There was no difference in hospitalization or mortality reported with CVAEs when stratified by type of therapy. On longitudinal follow‐up, overall cardiovascular events were significantly associated with combination therapy versus monotherapy when accounting for competing risk of death. Collectively, both cohorts demonstrated a higher tendency for heart failure and new or worsening hypertension as events, particularly in those receiving combination therapy compared to monotherapy in a “real‐world” setup, thus justifying a reason to follow them closely by cardio‐oncology during their therapy.

HF was noted to be significantly associated with combination therapy in the FAERS registry but not in the Truven Health Analytics/IBM MarketScan registry. In the meta‐analysis by Mincu et al., the relative risk of LVEF decline was 3.72.^11^ It should be recognized in our study, we specifically evaluated for symptomatic heart failure, not just EF declines which can often be asymptomatic and not associated with clinical heart failure. As such, our lower OR of 1.62 for HF with dual therapy is expected and consistent with prior findings. Most HF events were noted in the first 6 months in the combination arm. In the 5‐year follow‐up analysis of the COmbined LGX818 (encorafenib) used with MEK162 (binimetinib) in BRAF mutant Unresectable Skin cancer (COLUMBUS) trial,^17^ LVD events were noted mostly in the first 6 months (5.1%) compared to 6–12 month (3.4%), 12–18 month (3.4%) and 18–24 month period (1.7%).

Similar to HF, HTN was also significantly higher in the combination therapy arm in the FAERS registry but not in the Truven Health Analytics/IBM MarketScan registry. Apart from being identical to the meta‐analysis,^11^ this data relates well with the data presented in the 5‐year analysis of the COMBI‐V and COMBI‐D trial, which showed that 29% of patients had a hypertension event.^18^ In another 3‐year safety study of the COMBI‐V trial, HTN was noted in 24% of those with combination therapy compared to 16% in those receiving monotherapies.^6^ In another study comparing vemurafenib versus vemurafenib and cobimetinib combination therapy in those with metastatic melanoma (coBRIM), significantly higher HTN was noted in the combination therapy arm (15.4%) compared to the control arm (8.1%).^19^ These data suggest that attention should be paid to blood pressure in patients initiated on monotherapy or combination therapy as hypertension has been implicated in the risk of stroke, heart failure, and coronary artery disease in patients with cancer.^20^

The knowledge of the significant cardiovascular toxicity profile of these combinatorial regimens highlighted in our study and others^21^ is vital to anticipate specific off‐target CVAE and thus screen accordingly and take effective measures early in treatment, including cardiology referral for co‐management to prevent cardiovascular toxicity and unnecessary treatment disruption. Similar to other cytotoxic and targeted therapies, there may be a role for early surveillance with imaging, or biomarkers may be necessary early after combination therapy initiation. Studies have shown that 12% of patients with a normal LVEF at the completion of anthracycline‐based chemotherapy with ultimately develop dysfunction in subsequent years.^22^ As such, troponin monitoring with each cycle therapy is recommended to identify those at risk for cardiotoxicity and allow for intervention before permanent damage has occurred.^23^, ^24^ Similarly, regular monitoring for LVEF and global longitudinal strain changes may help minimize trastuzumab‐related cardiac dysfunction, which can approach 25% when used sequentially with anthracyclines.^23^, ^25^, ^26^

The pathophysiology of BRAF and MEK‐associated cardiotoxicity remains uncertain though various hypotheses have been suggested based on data from basic and translational studies.^27^, ^28^, ^29^ There is evidence to suggest that the MAPK pathway may be cardioprotective; BRAF and MEK inhibitors inhibit this pathway and can lead to hypertrophy, apoptosis, cardiac remodeling, and declines in LVEF as well as arterial hypertension via effects on nitric oxide production. Similarly, hypertension may result from BRAF and MEK effect on the renin‐angiotensin system.

Multiple limitations should be acknowledged. First, this is a retrospective pharmacovigilance study from a public registry and a retrospective cohort. The FAERS data is voluntarily reported by both patients and the medical community and requires no confirmation or scientific rigor, which leads to potential reporting and attribution biases. Truven Health Analytics/IBM MarketScan database does not contain a random sample of patient claims data but rather a cohort primarily drawn from large employers. Patients who are self‐insured and those insured through small and medium employers are underrepresented in the dataset. Similarly, patients covered only by public insurance (e.g., Medicaid, traditional Medicare, and Medicare Advantage) could not be included; however, using two datasets helped overcome that limitation somehow. FAERS data lack temporality due to its cross‐sectional nature, but we addressed that issue with the Truven Health Analytics/IBM MarketScan dataset. Even though FAERS lacked prior cardiovascular conditions, vital signs, and laboratory values including troponin or brain natriuretic peptide, electrocardiogram, echocardiogram or advanced cardiovascular, or oncologic diagnostic workup, Truven Health Analytics/IBM MarketScan was able to provide a significant number of these covariates, thus helping study profile of these patients. However, the Truven Health Analytics/IBM MarketScan dataset relies heavily on coders’ accuracy and is not comprehensive of all provider and patient characteristics. Truven Health Analytics/IBM MarketScan database did not have a sufficient number of patients indicating a lack of power to achieve statistical significance. Stated differently, some of the CVAEs had very low incidence, thus risking a significant likelihood of type 2 error. Additionally, BRAF/MEK combination therapy has a much longer progression‐free survival than single‐agent BRAF inhibitor, which leads to a more prolonged exposure that could partly account for the added toxicity. We could not account for the length of exposure in either data sets in a reliable manner. Lastly, the FAERS registry remains prone to issues related to all passive surveillance reporting systems, such as under‐, over‐, and duplicate reporting (despite efforts to exclude duplicate reports), varying report quality, and absence of denominator data.^30^, ^31^, ^32^ Accordingly, it is possible more cardiac adverse events may have gone uncaptured.

## 5 CONCLUSION

In these pharmacovigilance analyses spanning eight years and two large datasets, combination BRAF/MEK inhibitors were associated with increased CVAEs compared to BRAF monotherapy. Heart failure and hypertension are the most common events in this “real world” analysis, thus justifying a reason to follow them closely by cardio‐oncology during their therapy.

## CONFLICT OF INTEREST

None.

## ETHICAL APPROVAL

FAERS data are publicly available and does not require approval by the Institutional Review Board. Truven Health Analytics/IBM MarketScan dataset is de‐identified, an exemption was obtained from the University Hospitals’ Institutional Review Board.

## Supporting information

Supplementary Material

## Data Availability

The data that support the findings from the FDA’s Adverse Events Reporting System (FAERS) part of this study are openly available in raw format at https://bit.ly/37cHoXC. The data that support the findings of the Truven Health Analytics/IBM MarketScan part of the study are available from IBM. Restrictions apply to the availability of these data, which were used under license for this study. Thus, this data cannot be shared directly by the authors.
