# Supplementary material for: Cardiovascular adverse events associated with BRAF versus BRAF/MEK inhibitor: Cross‐sectional and longitudinal analysis using two large national registries
Source: Cancer Med. 2021 May 13;10(12):3862–72. doi: 10.1002/cam4.3938 (PMC8209554; doi:10.1002/cam4.3938)

**Supplemental/Appendix**

**Supplemental Methods:**

**Adjudication of Death in Truven Health Analytics/IBM MarketScan**

Prior to 2016, death was defined using the label of “20” for death in inpatient claims, “40” for expired at home based on hospice claims, “41” for expired in medical facility on hospice claims. Beginning in the 2016 data year, values indicating death were no longer used to protect patients' privacy. The disposition was set to missing. Hence, enrollment in/discharge to hospice with label of “50”/”51” along for labels “40” and “41” described above were considered to be death. Additionally, if there was a missing disposition with a palliative care service code determined by ICD-9 CM code of V66.7 or ICD-10 CM code of Z51.5 then the patient was considered to have died. The date of all the claims and labels mentioned here was considered to be the date of death.

**Supplemental table 1:** ICD-9/ICD-10 codes

| **Covariate/Adverse Event** | **Truven Health Analytics/IBM MarketScan** | | **FAERS** |
| --- | --- | --- | --- |
|  | **ICD-9 CM code** | **ICD-10 CM Code** | **ICH E2B key words** |
| Heart Failure | 398.91, 425.4x, 425.5x, 425.7x, 425.8x, 425.9x, 428.xx | I09.9x, I11.0x, I13.0x, I13.2x, I25.5x, I42.0x, I42.5x, I42.9x, I43.xx, I50.xx, P29.0x | Acute Left Ventricular Failure, Cardiac Dysfunction, Cardiac Failure, Cardiac Failure Acute, Cardiac Failure Congestive, Cardiogenic Shock, Cardiomyopathy, Cardiotoxicity, Congestive Cardiomyopathy, Cytotoxic Cardiomyopathy, Diastolic Dysfunction,  Dilatation Ventricular, Ischaemic Cardiomyopathy, Left Ventricular Dysfunction, Left Ventricular Failure, N-Terminal Prohormone Brain Natriuretic Peptide Increased, Right Ventricular Failure, Stress Cardiomyopathy, Tachycardia Induced Cardiomyopathy, Ventricular Dysfunction |
| Ischemic Stroke | 430.xx – 438.xx OR procedure codes:  00.61, 00.62, 00.63, 00.65, 38.12, 38.32, 38.42, 39.22, 39.28, 39.74 | G45.xx, G46.xx, H34.0x, I60.xx - I69.xx | Embolic Cerebral Infarction, Embolic Stroke, Ischaemic Stroke, Transient Ischaemic Attack |
| Myocardial Infarction | 410.xx | I21.xx, I22.xx | Acute Myocardial Infarction, Angina Unstable, Myocardial Infarction, Myocardial Ischaemia, Myocardial Necrosis Marker, Troponin I Increased, Troponin Increased, Troponin T Increased |
| Pulmonary Embolism | 126.0x, 415.1x, 634.6x, 635.6x, 636.6x, 637.6x, 638.6x, 639.6x, 673.2x | I26.9x | Pulmonary Embolism |
| Deep Vein Thrombosis | 453.2x, 453.4x | I82.2, I82.4 | Deep Vein Thrombosis |
| Venous Thromboembolism | DVT+PE above | DVT+PE above | DVT + PE +  Axillary Vein Thrombosis, Hepatic Vein Thrombosis, Jugular Vein Thrombosis, Portal Vein Thrombosis, Subclavian Vein Thrombosis, Venous Occlusion, Venous Thrombosis |
| Arterial Hypertension | 401.xx | I10.xx | Accelerated Hypertension,  Blood Pressure Increased,  Blood Pressure Systolic Increased,  Diastolic Hypertension,  Essential Hypertension,  Hypertension,  Hypertensive Crisis,  Hypertensive Heart Disease,  Hypertensive Urgency,  Labile Blood Pressure,  Systolic Hypertension |
| Diabetes | 250.xx, 362.xx | E10.0x, E10.lx, E10.6x, E10.7x, E10.8x, E10.9x, E11.0x, E11.1x, E11.6x, E11.7x, E11.8x, E11.9x, E12.0x, E12.1x, E12.6x, E12.7x, E12.8x, E12.9x, E13.xx, E14.xx |  |
| Atrial Fibrillation | 427.3x | I48.xx | Atrial Fibrillation,  Atrial Flutter |
| QT prolongation |  |  | Electrocardiogram Qt Prolonged, Torsade De Pointes |
| Melanoma | 172.xx | C43.xx | Melanoma |
| Non-Small Cell Lung Cancer | 162.xx | C33.xx,C34.xx | Lung Cancer |
| Colon Cancer | 153.xx | C18.xx | Colon Cancer |
| Other Cancer | 140.xx – 209.xx (excluding above) | C00.xx - C96.xx (excluding above), C7A.xx |  |
| **Procedures** | **CPT Code** | |  |
| Anthracycline Use | J9000, J9001, J9010, J9178, J9180 | |  |
| Radiation therapy | 77401, 77402, 77407 , 77412, 77371, 77372, 77373, 77778, 0394T, 0395T, 77424, 77425, 77789, 77750, 77790, G6001-G6017, 77385 -77387, 77520 – 77525, 77422 – 77423, 77761 – 77763, 77600 – 77620, 77385 – 77387, 77770 - 77772 | |  |

**Supplemental figure 1 A-C:** Competing risk analysis of (A) stroke event, (B) venous thromboembolism event, and (C) atrial fibrillation in those receiving MEK and BRAF combination therapy vs. monotherapy from Truven Health Analytics/IBM MarketScan dataset


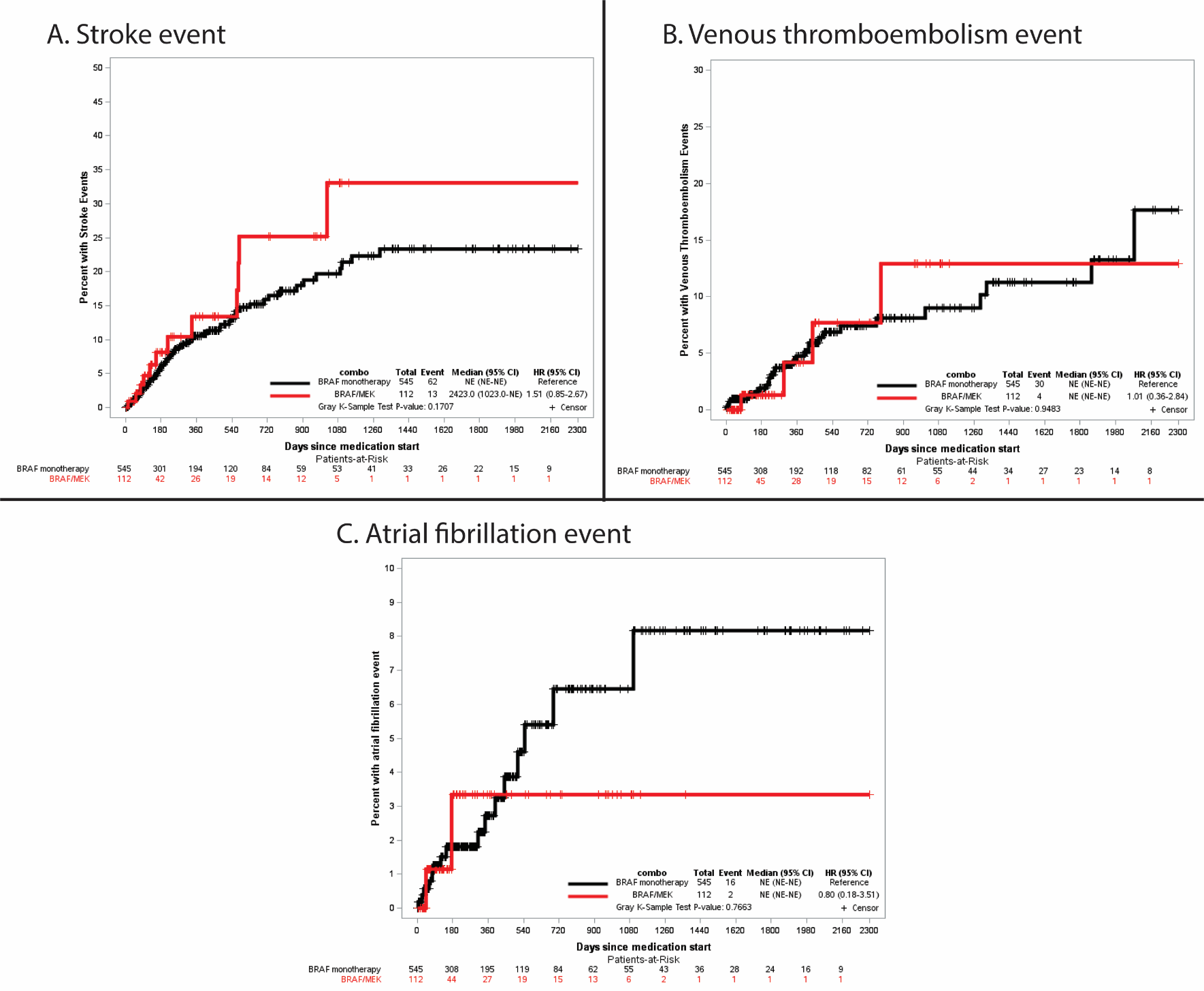

Supplement: Supplementary file 1 — Supplementary Material [file CAM4-10-3862-s001.docx]
